# Ingestion Rate, Prey Selectivity, and Growth of Larval *Vieja zonata* (Teleostei: Cichlidae) Co-Fed Rotifers with Cladocerans

**DOI:** 10.1155/2024/6424063

**Published:** 2024-07-24

**Authors:** Rubén Alonso Contreras-Tapia, Gabriela Garza-Mouriño, María Elena Castellanos-Páez, Manuel Castillo-Rivera, Nandini S., Marcela Ivonne Benítez-Díaz Mirón

**Affiliations:** ^1^ Laboratorio de Rotiferología y Biología Molecular de Plancton Departamento El Hombre y su Ambiente Unidad Xochimilco Universidad Autónoma Metropolitana, Ciudad de México C.P. 04960, Mexico; ^2^ Laboratorio de Peces Departamento de Biología Unidad Iztapalapa Universidad Autónoma Metropolitana, Ciudad de México C.P. 09310, Mexico; ^3^ Laboratorio de Zoología Acuática Universidad Nacional Autónoma de México, Campus Iztacala, Estado de México C.P. 54090, Mexico

## Abstract

The study of fish larval nutrition is important as dietary requirements change significantly with growth. *Vieja zonata* is a cichlid species that is endemic to Mexico. In this study, we investigated the ingestion rate, prey selectivity, and growth of *V. zonata* larvae that were fed on prey cultured with and without probiotics. We conducted three experiments to test the acceptability of the prey offered, determine the optimal density of the prey items, and observe the effect of probiotics on the larvae's growth. The first experiment tested the acceptability of the prey offered to 5 days post-hatched (dph) *V. zonata* larvae, and the second experiment determined the optimal density of the prey items. In the third experiment, we individually placed 5 dph larvae (SL = 5.97 ± 0.13 mm; 8.5 ± 0.25 mg) and fed them for 10 days with three different prey items: two rotifer species (*Brachionus angularis* and *Plationus patulus* at a density of 20 ind/mL) and a cladoceran species (*Moina* cf. *macrocopa* at 1 ind/mL), both cultures with and without probiotics. We counted the prey items consumed daily and provided fresh media with new prey at the above density. We determined the total counts, ingestion rates, and Manly's selectivity index (*α_i_*) and measured and weighed the larvae at the beginning and end of the experiment. The endogenous feeding period with the yolk sac lasted until 5 dph, a mixed period with endogenous and exogenous feeding occurred from 5 to 7 dph, and an exogenous feeding period in which they fed on zooplankton was observed from day 7 to 15 dph. *Brachionus angularis* and *Plationus patulus* were accepted as prey after 5 dph, and *Moina* cf. *macrocopa* was accepted on 11 dph. During the first days of feeding, the preferred prey item was *P. patulus*, which later switched to *M*. cf. *macrocopa* on day 11. We found that the use of prey produced with NanoCrusta probiotics resulted in a significant increase in the somatic growth and weight of *V. zonata*. Our findings suggest that probiotics may potentially enhance the nutritional value of prey items and promote the growth of *V. zonata* larvae.

## 1. Introduction

The transition from endogenous to exogenous feeding in fish larvae is a critical period in their development, marked by high mortality rates under laboratory conditions and in the environment [1, 2]. During this stage, adequate prey availability is essential, as even minor alterations or starvation can significantly impact the intestinal microbiota structure and cause substantial morphological, physiological, and biochemical changes [3, 4, 5, 6]. Sufficient food supply is crucial for the development and growth of altricial species (e.g., cichlids), directly reducing starvation mortality during the first feeding period and indirectly affecting growth rates, which are vital for aquaculture. Besides that, prey density availability is essential because fish larvae react to increasing prey in two ways: (1) functional response: the number of prey consumed increases as prey density increases until larvae reach satiety; (2) numerical response: an increase in prey density supports better nutrition and survival of larvae which later can lead to higher reproduction rates in the reproductive stage [7]. Functional response experiments indicate appropriate food levels for fish at specific ages [8]. Therefore, the knowledge related to feeding during the larval stage is of great significance in aquaculture since this data will allow them to establish feeding regimes for successful rearing.

Cichlid larvae hatch with a digestive system that is neither well-developed nor fully functional, requiring external nutrient sources after vitellus absorption for proper development and growth [9, 10, 11]. To start feeding exogenously, the organs and structures involved during the detection, capture, ingestion, digestion, and assimilation of food must be functional and exogenous food available to be consumed by the hatchlings [2, 12]. The full anatomical development in some cichlids occurs after the start of exogenous feeding [9, 11]. Previous studies have reported that larvae are not capable of processing formulated diets [2, 6, 11], so live food is the best choice during the early developmental stages of fish [13, 14, 15]. Recent evidence indicates that commercial feeds can adversely affect growth and digestive tract morphology in early developmental stages in species of *Vieja* (e.g., *Vieja melanurus*) [11].

Along with digestive tract development, the community structure of the larval microbiota evolves during metamorphosis. Inadequate larvae–microbe interactions are a significant cause of poor performance during larval rearing [16]. A well-established intestinal microbiota provides multiple benefits including pathogen competition, immune system stimulation, nutritional factors provision, transcriptional effects, and metabolism improvement [16, 17, 18]. Probiotic bacteria, commonly used in aquaculture, enhance these benefits when added to rearing water or bioencapsulated in live food or formulated diets [19, 20]. Recent studies have highlighted the positive impact of probiotics on larval health and development, emphasizing their role in enhancing growth performance, survival rates, and immune responses in aquaculture. Probiotics may also stimulate appetite and improve nutritional quality, making them a valuable addition to larval diets [20, 21].

*Vieja zonata* (Meek, 1905), known as Mojarra prieta, is a cichlid endemic to Mexico that lives in rivers along the Isthmus of Tehuantepec in Oaxaca from Río Tequisistlán to Río Tapantepec and on the Atlantic slope in the Río Coatzacoalcos basin [22, 23, 24]. *V. zonata* can reach >28 cm SL and is a species with ecological and economic significance, with a growing interest in the national and international market for ornamental aquaculture. This species is caught and consumed by locals with a growing interest in production due to characteristics such as regular size, moderate growth, native, good taste, and acceptability. These characteristics make *V. zonata* suitable for production, as well as other native Mexican mojarras (*Mayaheros urophthalmus* [25, 26]). Understanding functional response and ingestion rates is essential to determine appropriate food levels for larval stages. Therefore, this study aimed to investigate the first feeding, prey selectivity, somatic growth, and weight of *V. zonata* larvae when fed live prey produced with probiotics and without probiotics. We hypothesized that the prey selectivity and the somatic growth of the fish larvae would be greater when consuming zooplankton enriched with probiotics and that *V. zonata* larvae would shift toward probiotic-enhanced zooplankton over time.

## 2. Materials and Methods

### 2.1. Breeding of *V. zonata* and Live Feed Culture

*V. zonata* pairs were kept in 240 L tanks with flagstone rocks but no substrate. The selection of these tanks was based on their capability to facilitate successful fish reproduction and simplify maintenance and handling processes. To ensure optimal conditions, the tanks had continuous aeration and mechanical, chemical, and biological filtration. A 12 : 12 hr photoperiod (light:dark) was maintained, and weekly maintenance involved replacing 10% of the tank water with treated tap water. The treated water underwent chlorination by adding 10 mg/L of NaClO for 30 min, then neutralized with Na_2_S_2_O_3_ · 5H_2_O, and aeration until use. Water parameters were pH = 8.0 ± 0.5, temperature = 25 ± 1°C, and dissolved oxygen (DO) = 6.0 ± 0.5 mg/L. *V. zonata* broodstock had been kept in laboratory conditions for at least 2 years and spawned regularly at the Laboratory of Rotiferology and Molecular Biology of Plankton at the Metropolitan Autonomous University, Xochimilco (UAM-X). The pairs were fed three times daily on a commercial diet (El Pedregal; 42% protein content). Throughout the spawning process, the fish were carefully monitored, and once completed, the time was considered as 0 hr. The fertilized spawn was attached to a rock and placed in a 40-L aquarium filled with water from the main aquarium, maintaining identical water conditions. Different spawns were obtained following the protocol described to characterize development associated with feeding, first feeding occurrence, and subsequent experimentation time. After hatching, the larvae were kept in the same aquarium. Five larvae were observed every 12 hr under a microscope to see development and yolk sac absorption. Pigmented eyes, mouth opening, and free swimming typically developed after 5 days post-hatch (dph), at which point the larvae were used in all experiments (SL = 5.97 ± 0.13 mm; 8.5 ± 0.25 mg; 670.74 ± 67.50 *µ*m gape size).

Three species of zooplankton were cultured as live feed separately in 1 L polypropylene containers with EPA medium [27] under controlled conditions (25 ± 1°C with artificial lighting under a 12 : 12 hr (L:D) photoperiod). The zooplankton species included *Brachionus angularis* (rotifer; 97.29 ± 5.9 body length (BL)), *Plationus patulus* (rotifer; 154.35 ± 19.6 *µ*m BL), and *Moina* cf. *macrocopa* (Cladocera; 1,227.94 ± 37.6 *µ*m BL). The preys were cultured with or without probiotics, and those without probiotics were fed with 1 × 10^6^ cells/mL of *C. vulgaris*, while those with probiotics were fed with 2.12 × 10^6^ cells/mL NanoCrusta probiotics + 1 × 10^6^ cells/mL *Chlorella vulgaris* [28]. The composition of NanoCrusta includes *Lactobacillus plantarum*, *L. casei*, *L. fermentum*, *L. delbrueckii*, *Bacillus subtilis*, and *Rhodopseudomonas palustris* at 2.12 × 10^9^ cells/mL. These probiotics were selected based on the previously reported favorable effects on zooplankton growth parameters [28]. Zooplankton was harvested daily before every feeding experiment by passing through a 55 *µ*m mesh for rotifers and a 210 *µ*m mesh for cladocerans. Densities were estimated by averaging three 1-mL aliquots for each species and quality under a Nikon SMZ2800 microscope.

### 2.2. Prey Acceptance and Optimum Prey Density

Two experiments were conducted to evaluate the functional response and optimal density of prey for *V. zonata* larvae. The first experiment aimed to assess prey acceptance at 5 dph. Six different treatments for each rotifer species, with densities of 1, 5, 10, 15, 20, and 25 rotifers per mL. The second experiment aimed to evaluate prey acceptance at 15 dph. Five different treatments were tested with densities of 0.2, 0.4, 1.0, 1.5, and 2.0 cladocerans per mL. All treatments had five replicates for every zooplankton species. For each experiment, five *V. zonata* larvae were placed individually in 120 mL polypropylene vessels with 50 mL of water. The larvae were starved for 1 hr before experiments. The vessels were kept at a constant temperature of 25 ± 1°C in a water bath. Freshly harvested prey was offered at the described density, and after 60 min, the remaining prey was counted to determine the ingestion rate (*I*_*R*_; prey/larvae/hour). The ingestion rate was calculated by comparing the initial and final prey densities using Equation (1) described by Caldeira [29]:

(1) $$\begin{array}{c} IR=\left[\left(Ci-Cl\right)\times \left(Ci-Cf\right)/n\right]/t, \end{array}$$

where *I*_*R*_ is the ingestion rate (prey/larvae/hour), *C*_*i*_ is the initial density of prey, *C*_*f*_  is the final density of prey, *C*_*l*_ is the final prey concentration in the control beakers, *n* is the number of larvae, and *t* is the duration of the experiment (hours).

### 2.3. Ingestion Rate, Prey Selection, and Growth of *V. zonata* Larvae Fed with Two Prey Qualities

Ingestion rates, prey selection, and growth of larvae during the first 10 days of exogenous feeding were compared with zooplankton cultured with and without probiotics. To this end, one 5-dph larva was placed individually in 120 mL polypropylene vessels filled with 80 mL of water, randomly selected from the same spawn.

The vessels were aerated using a fine-tip modified Pasteur pipette with constant bubbling and were maintained at 25 ± 1°C by placing them in a water bath. The experimental design included five treatments, comprising: (1) probiotic-free prey without larvae (VzC01); (2) probiotic-enhanced prey without larvae (VzC02); (3) larvae without prey (VzC03); (4) larvae fed probiotic-free prey (VzC04); and (5) larvae fed probiotic-enhanced prey (VzC05). All treatments had seven replicates. Each rotifer species was offered together at a density of 10 ind/mL (a total of 20 rotifers/mL) and cladocerans at 1 ind/mL, in addition to *C. vulgaris* at 0.5 × 10^6^ cells/mL. The selected prey densities were determined based on the results of the functional response experiment mentioned earlier. Treatments without larvae were used as controls to estimate the increase in prey density due to reproduction, and densities were adjusted to the initial treatment density every 24 hr. In treatments with larvae fed on prey, freshly harvested prey of each quality was offered every 24 hr at the density described. The remaining prey was preserved in 4% formalin and the total count was conducted using a microscope. The number of prey items consumed was used to estimate the *I*_*R*_ and Manly's *α* selectivity index. The experiment was stopped when the predator consumed almost 100% of the prey offered. The Manly's *α* selectivity index (*α_i_*) was calculated using Equation (2):

(2) $$\begin{array}{c} \alpha_{I}=\log p_{i}/\sum_{j=1}^{m}p_{j}, \end{array}$$

where *α_i_* is the Manly's *α* (preference index) for prey type *i*; *pi*, *pj* is the proportion of remained prey *i* or *j* remaining at the end of the experiment (= *e*_*i*_*/n*_*i*_); *ei* is the number of remained prey type *i* remaining uneaten at the end of the experiment; *ni* is the initial prey density type *i* in the experiment; and *m* is the number of prey types [30]. For this index, the number of prey types is considered, and the following criteria are used when: (1) *α_i_* = 1/*m*: the feeding is not selective; (2) *α*_*i*_ > 1/m: the predator prefers the prey species *i* in its diet; *α*_*i*_ < 1/m: the prey species *i* is avoided. Since three prey items were offered in the experiment 1/m = 0.33 [30].

Standard length (SL), body depth (BD), and weight (W) were determined at the beginning and end of the experiment. Somatic growth of the larvae was estimated using digital image analysis. Images were captured using a Nikon D3400 SLR camera with a Nikkor micro 55 mm 1 : 28 lens and a Nikon NI-30 fiber optic illuminator. Images were calibrated using an Olympus 1-mm calibration ruler and processed for measurement using ImageJ 1.51S software (NIH, USA). To weigh the larvae, each one was individually weighed using an Ohaus microbalance (EP214C, *d* = 0.1 mg). The specific growth rate (SGR) was estimated based on SL and BD using following Equation (3):

(3) $$\begin{array}{c} \text{SGR}\left(\%\right)=\left[\frac{\log\;\left({\text{SL}}_{f}\right)–\log\;\left({\text{SL}}_{i}\right)}{t}\right]\times 100, \end{array}$$

where SGR is the specific growth rate, SL is the standard length, *f* is the final, *i* is the initial, and *t* is the time (days) [31].

All procedures were performed adhering to strict standards for animal care and research set by the Mexican government (NOM-062-ZOO-1999) and “The International Council for Laboratory Animal Science (ICLAS).”

### 2.4. Data Analysis

All values are expressed as mean ± standard deviation (SD) and standard error of the mean (SE) in the figures. Initially, to analyze the functional response, a logarithmic regression model was applied (*y* = *b* + *a* × ln (*x*), where “*x*” denotes prey density, “*y*” represents the ingestion rate, “ln” is natural logarithm, and “*a*” and “*b*” are estimated regression coefficients. Where this model reaches the asymptotic value is the point considered as optimal prey density, which is used in the next experiment. The significance of this model was evaluated using a one-way analysis of variance (ANOVA).

To determine significant differences between treatments for variables such as ingestion rates, prey selectivity, somatic growth, and weight, we performed ANOVA. This analysis was followed by Tukey's honestly significant difference post hoc test to identify specific group differences. The statistical software Past 3.20 was used for these analyses [32, 33].

For prey consumption and prey selectivity data, we employed a repeated measures ANOVA design. This design included one between-subjects factor (treatments) and one within-subjects factor (time), allowing us to examine how prey consumption and selectivity changed over time and across different treatments. Following the repeated measures ANOVA, we conducted a Tukey's post hoc test for multiple comparisons to pinpoint specific differences between groups over time. Before conducting all these tests, we assessed the assumptions of normality (D'Agostino–Pearson), homoscedasticity (Levene), and independence (Durbin–Watson) [32].

## 3. Results

### 3.1. Prey Acceptance and Optimum Prey Density

**Figure 1:**
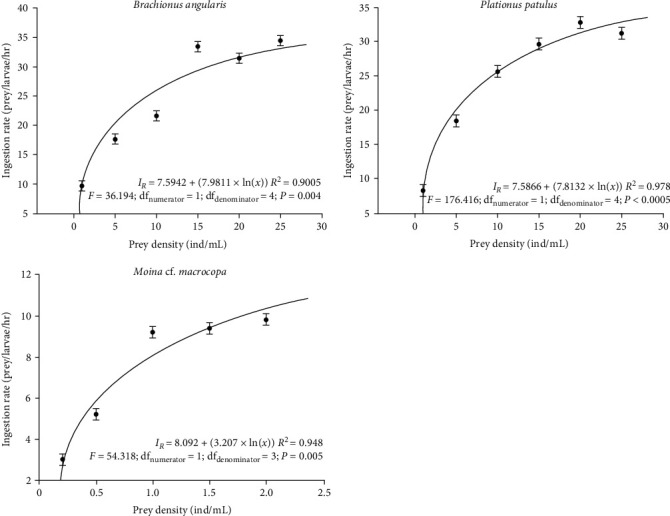
*V. zonata* larval ingestion rates in response to increased prey availability. The mean is shown based on the five replicates of each treatment and the logarithmic regression model *y* = *b* + *a* × ln(*x*).

The functional response curves indicate that *V. zonata* larvae accepted all three zooplankton species as prey. At 5 dph, larvae consume between 9.6 and 34.4 rotifers/larvae/hour when fed with *B. angularis* compared to 8.2–32.8 rotifers/larvae/hour when *P. patulus* was offered as prey (Figure 1). ANOVA analysis showed significant differences between ingestion rates on *B. angularis* and *P. patulus* (*P*  < 0.001). An increase in the ingestion rate was associated with increasing prey availability; however, no significant differences were found when *B. angularis* was offered from 15 to 25 rotifer/mL (Tukey *P*  > 0.05). Similarly, ingestion rates were not significantly different for larvae fed with *P. patulus* from 15 to 25 rotifer/mL (Figure 1; Tukey *P*  > 0.05).

As prey density increased, the ingestion rate of *M*. cf. *macrocopa* at 15 dph increased (Figure 1). Larvae consumed from 3 to 9.8 cladocerans/larvae/hour. Ingestion rates showed significant differences among treatments (ANOVA; *P*  < 0.001); nonetheless, no differences were observed between treatments fed from 1.0 to 2.0 cladocerans/mL (Figure 1; Tukey *P*  > 0.05).

### 3.2. Ingestion Rate, Prey Selection, and Growth of *V. zonata* Larvae Fed with Two Prey Qualities

**Figure 2:**
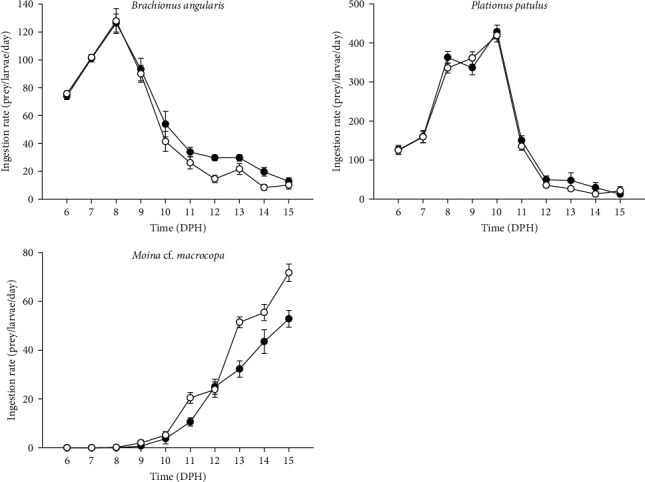
Larval ingestion rates of *V. zonata* during the first 10 days of exogenous feeding. The mean ± SE is shown. Larvae fed with probiotic-free prey (solid black dots); larvae fed with probiotic-enhanced prey (open dots).

**Figure 3:**
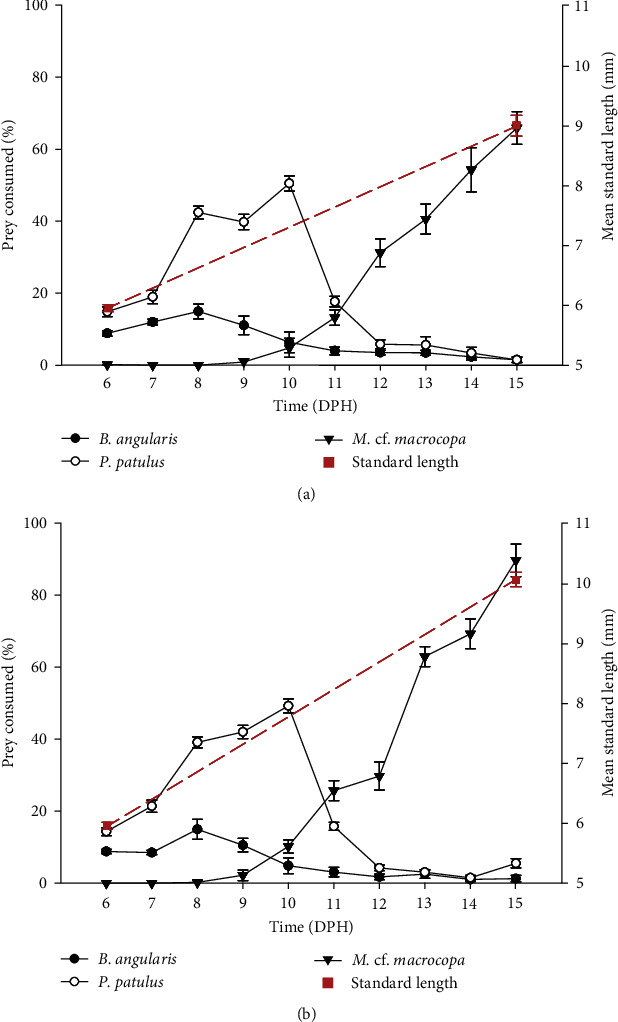
Percentage of prey consumed from 5 to 15 dph by *V. zonata* larvae fed with the prey of two qualities. The mean ± SE for each day based on the seven replicates is shown: (a) larvae fed with probiotic-free prey; (b) larvae fed with probiotic-enhanced prey.

*V. zonata* larvae fed on rotifers and cladocerans from 5 to 15 dph. Larval ingestion rates are shown in Figure 2, while the percentage of prey consumed is presented in Figure 3. From 5 to 10 dph, larvae fed mainly in rotifers with a strong preference toward *P. patulus* in both treatments with live prey, then from day 11 to 15 dph larvae switched to *M*. cf. *macrocopa* as the main resource, which was consistent on both treatments. The highest consumption of rotifers was >400 rotifers/larvae/day, and the highest consumption of cladocerans was 70 cladocerans/larvae/day. On the sixth experimental day (11 dph) larvae changed their preference from rotifers to cladocerans for both treatments. On 13 dph, one of the replicates from the treatment of larvae fed on probiotic-free prey died, while in the treatment fed on probiotic-enhanced prey, all the replicates survived until the end of the experiment.

By expressing the consumption as a percentage of prey consumed, it was possible to compare rotifers and cladocerans consumption. For both treatments, day 11 dph represented a change in prey consumption from rotifers to cladocerans. The larvae fed with probiotic-enhanced prey consumed close to 100% of the prey on 15 dph (89.62% ± 4.50%), compared to 65.89% ± 4.58% on the probiotic-free treatment. The slope of the growth of the standard length was greater in the treatment fed with probiotics (slope: 0.45 vs. 0.33; Figure 3). At the end of the experiment, the probiotic-enhanced treatment had 100% survival, while probiotic-free treatment had 85.7%.

Repeated measures ANOVA revealed a significant effect of zooplankton quality in *B. angularis* and *M*. cf. *macrocopa* consumption by larvae (ANOVA; *P*=0.036 and *P*  < 0.0002, respectively), while *P. patulus* showed only marginal differences (*P*=0.08). The treatment × time interactions were significant (ANOVA; *P*  < 0.05), indicating that the treatment effect depends on time. In rotifers, non-enhanced prey tends to be consumed more, but with cladocerans, more probiotic-enhanced prey are consumed.

**Figure 4:**
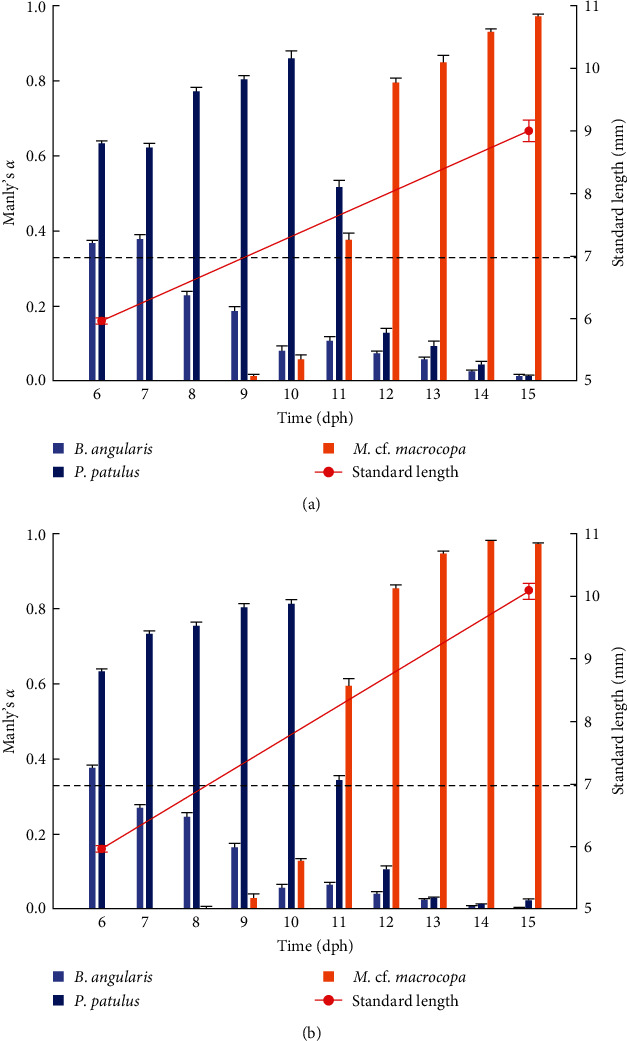
Prey selectivity (Manly's *α*) by *V. zonata* larvae fed on three different prey items and of two qualities (probiotic-free and probiotic-enhanced) during the first 10 days of exogenous feeding. The mean ± SE is shown. The dotted line represents the *αi* (*αi* = 0.33): (a) probiotic-free prey; (b) probiotic-enhanced prey.

*V. zonata* larvae exhibit changes in food preferences throughout the days (Figure 4). The Manly's *α* index confirmed a strong preference of larvae toward rotifers during first the 5 days preceded by a change in the preference toward cladocerans. Results showed that *B. angularis* was not preferred while *P. patulus* was the preferred prey for larvae for both experimental treatments during the first days. From 11 dph, the cladoceran *M*. cf. *macrocopa* was the preferred prey. The main difference between treatments took place on 11 dph, for probiotic-free treatment *P. patulus* was the preferred prey over *M*. cf. *macrocopa*, in contrast to probiotic-enhanced treatment where *M*. cf. *macrocopa* was the preferred prey over *P. patulus*. For the three prey species, repeated measures ANOVA indicated statistically significant differences between treatments and time on prey selectivity for *V. zonata* (ANOVA; *P*  < 0.05; except for *P. patulus* *P*=0.1057).

**Figure 5:**
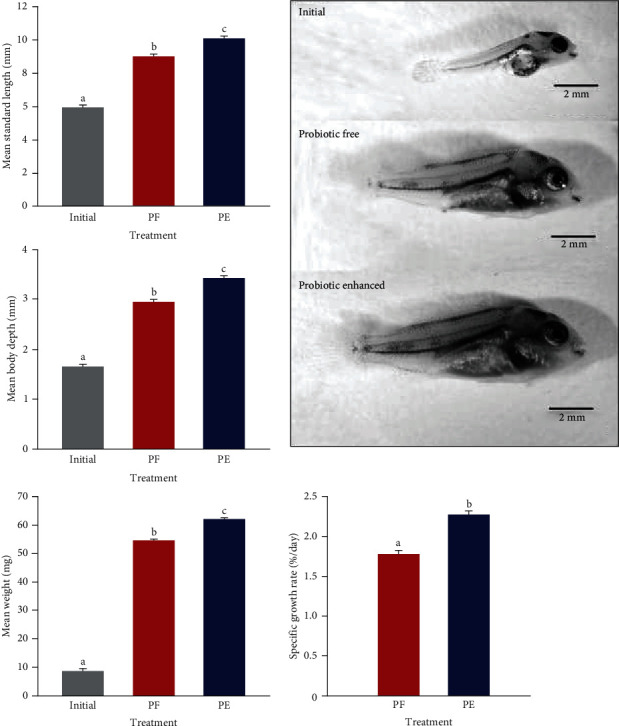
Somatic growth, weight, and specific growth rate of *V. zonata* fed on different prey quality. The mean ± SE is shown. For each variable, the data that have the same letter do not present significant differences (*P*  < 0.05, Tukey's pairwise comparisons test). PF, probiotic-free treatment; PE, probiotic-enhanced treatment.

Probiotic-enhanced treatment showed significantly higher meristic parameters (SL and BD) compared to probiotic-free treatment (ANOVA; *P*  < 0.001; Figure 5). Larvae fed on probiotic-enhanced preys were 12% greater in SL (10.08 ± 0.33 vs. 9.00 ± 0.85 mm) and 16.2% greater in BD (3.43 ± 0.14 vs. 2.95 ± 0.31 mm). The initial weight of 5-dph *V. zonata* larvae was 8.5 ± 0.25 mg. Probiotic-enhanced treatment also increased the weight of larvae compared to probiotic-free treatment. The larvae fed on probiotic-enhanced prey weighed 62.02 ± 1.81 mg, which was 13.5% higher than the probiotic-free treatment (54.60 ± 2.43; Figure 5). Probiotic-enhanced treatment yielded a significantly higher specific growth rate (2.27 ± 0.14%/day), compared to probiotic-free treatment (1.78 ± 0.21%/day; Figure 5; ANOVA; *P*  < 0.001).

**Figure 6:**
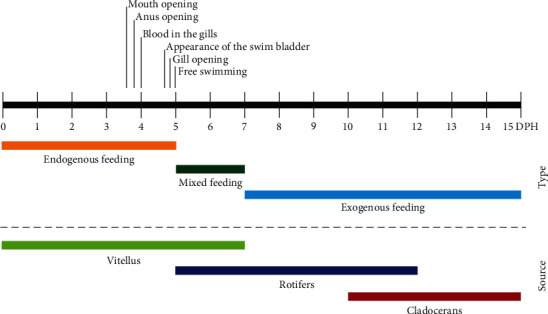
Feeding regime for *V. zonata* larvae during the first 15 dph.

*V. zonata* larvae can feed exogenously even when they have a yolk sac (endogenous feeding), which shows that they have a mixed diet during their larval stage. The mouth opening and anal opening occurred between days 3 and 4 after hatching; however, prey consumption at this stage is improbable. The feeding regime accompanied by some key developmental features for the transition from endogenous to exogenous feeding of *Vieja zonata* is shown in Figure 6. The consumption of rotifers is considered between day 5 and 12 dph, while the consumption of cladocerans was from 11 to 15 dph. During day 11, the change in prey selectivity occurred, despite this, the consumption of rotifers continued during day 12.

## 4. Discussion

In the past few decades, world inland-water fishing has increased by over 60% [34]. Historically, native Mexican freshwater fishes were utilized by prehispanic cultures and remain vital today, with species like cichlids, poecilids, atherinids, characids, and various catfish families being significant protein sources in rural Mexican communities [35, 36, 37, 38]. Lyons et al. [38] stated that 23.9% of Mexican freshwater fish contribute to subsistence fisheries, 18.3% to local commercial fisheries, 26.5% to national fisheries, and 14.6% to international fisheries. However, overharvesting and the introduction of non-native species significantly impact natural populations, with fishing recognized as a major threat [38]. Native fish, especially cichlids, are ideal for aquaculture due to their size, white meat, good taste, and acceptability. Understanding their basic biology and nutritional needs is essential for successful captive production [9, 25, 39].

Early development is important in altricial species because development continues post-hatching. Feeding in the larval stage is crucial because poor nutrition leads to serious development problems, with significant morphological, functional, and biochemical changes, and even death [11, 16, 40]. While much is known about the early ontogeny of *Danio rerio* [41], there is a limited information on Middle American cichlids. For cichlid species, studies are limited to a few species, such as *Oreochromis niloticus* [42]. Reports for Middle American cichlid species are scarce: *Mayaheros urophthalmus* [9, 43], *Amphilophus trimaculatus* [44], but no reports for species of the genus *Vieja*. Our study contributes crucial information by providing insights into the early ontogeny and feeding behavior of *V. zonata*, a Middle American cichlid.

The critical morphological changes for successful larval feeding are little known for most species [40, 45]. *V. zonata* hatched at 60 hr postfertilization which is comparable to other cichlid species such as *Amphilophus xiloaensis* [10] and *Cichlasoma dimerus* [46]. Some ontogenetic events are significantly related to the ability to feed exogenously (e.g., mouth-opening and free swimming). In a successful transition between endogenous and exogenous feeding, the coordinated structures and organs for food detection, capture, ingestion, digestion, and assimilation must be well developed, and live prey must be available [2]. The mouth-opening of *V. zonata* larvae began at 3 dph with free swimming until 5 dph when the gape size was 670.74 ± 67.50 *µ*m. In other cichlid species like *A. xiloaensis* [10], *C. dimerus* [46], and *Herichthys minckleyi* [47], mouth-opening occurs between day 2 and 3 dph and free-swimming also occurs on the day 5 dph.

At 5 dph, *V. zonata* larvae can feed exogenously, and by day 15, morphological changes were visible (Figure 5). The larval stage in fish begins from hatching, encompassing (1) the time when newly hatched larvae carry their yolk sac as an endogenous food source and (2) the larval stage after yolk sac absorption and subsequent exogenous feeding, and before metamorphosis to its juvenile form [4, 48, 49]. *V. zonata* larvae have a mixed feeding type during early post-hatching days, represented by an endogenous feeding type with vitellus (from 0 to 5 dph), followed by a mixed-feeding time (from 5 to 7 dph) and subsequently an exogenous feeding (from 7 dph; Figure 6). Mixed feeding, defined as an overlap of endogenous and exogenous feeding, is characteristic of underdeveloped larvae [50]. *V. zonata* has two crucial feeding points during early development: (1) the transition from endogenous to exogenous feeding, characterized by a short-mixed period, and (2) the change from small prey (rotifers) to larger prey (cladocerans; 11 dph). These moments are crucial because the absence of adequate food drives larval development issues and high mortalities, a principal problem in larval rearing [51]. During mixed feeding, it is likely that endogenous feeding is sustained as a backup while the larvae learn to acquire their food exogenously or when prey are deficient [50, 52]. Therefore, it is a flexible stage since, if the exogenous food is not present or available, then endogenous feeding continues, but it can have a direct consequence on the survival of larvae. When the vitellus is exhausted without exogenous food present, starvation causes a delay in digestive tract development with progressive deterioration and muscle formation issues [16, 45, 53, 54] combined with the risk of reaching the point of no return [16, 40, 45, 54]. Nutritional deficiencies caused by starvation are because the energy needed for maintenance, development, and growth is not enough, with a chain reaction that diminishes survival [45, 55].

One of the most common methods to assess prey consumption by fish is the acceptance tests that measure the number of prey items ingested and consumed by a predator [16, 56]. *V. zonata* larvae can detect, capture, consume, and ingest successfully the two rotifer species: *B. angularis* and *P. patulus* (from first feeding) and *M*. cf. *macrocopa* (from 11 dph; Figure 2). Successful feeding depends on elementary aspects of fish biology: morphology and physiology [2, 57].

The ingestion rate of *V. zonata* larvae at higher prey density reached an asymptote level corresponding to type II functional response. This type of response is characterized by a rapidly increasing number of prey items consumed by the predator, although it then stagnates despite the increase in prey density [58, 59], indicating that satiation has been reached. Type II functional response is described for other cichlid species (e.g., *O. niloticus* [60, 61]).

From an ecological perspective, the feeding behavior of *V. zonata* larvae might provide an ecological advantage, allowing them to avoid the negative consequences of overfeeding and thereby enhancing their survival prospects. In contrast, *Maskaheros regani* larvae, a syntopic species to *V. zonata*, display a voracious appetite with a feeding frenzy that leads to overconsumption and mortality (unpublished-own data). Appetite in fish is controlled by a complex interplay of endocrine signals between central (brain) and peripheral (intestine) systems [15, 62, 63]. Satiety signals have a major impact on appetite and meal size [15]. In the case of *M. regani*, the lack of effective satiety regulation leading to overfeeding can be contrasted with the potentially more balanced feeding behavior of *V. zonata*. This difference in feeding strategies may lead to higher survival rates for *V. zonata*, influencing species dynamics and niche differentiation within their environment. Further studies should investigate the endocrine regulation of feeding, survival rates, resource partitioning, environmental impacts, and evolutionary pressures to understand the feeding behaviors and ecological interactions of larvae.

The results suggest that the consumption of prey by larvae is determined by age and prey quality. The range of accessible particle sizes increases with the larvae size. During the larval stage, the morphological and physiological changes require greater consumption of food, the daily ingestion rate increases, and results in size and weight gain [2, 64, 65]. *V. zonata* larvae consumed fewer probiotic-enhanced rotifers compared to the probiotic-free treatment. This observation suggests that during the initial days, the larvae may have reached satiety more quickly by ingesting fewer rotifers, potentially due to the addition of probiotics. Later, the larvae consumed more probiotic-enhanced prey (cladocerans). This could be due to the probiotics having possible appetite-stimulating properties [66], or it might be because as the larvae grow larger, they might prefer prey with potentially higher nutritional value [15]. The nutritional quality of the probiotic-enhanced prey was evident in the increase in the specific growth rate (SGR; Figure 5). The increase in SGR using probiotics corresponds with previous reports for other fish species (e.g., *Oreochromis mossambicus* [67, 68, 69]).

Despite the differences in size, shape, and swimming pattern in rotifer species [70, 71], larval ingestion rate was similar for both rotifer species when prey items were offered alone (Figure 2). Larvae co-fed with both rotifer species showed a strong preference for *P. patulus* (Figure 3). Food selection by fish larvae depends on prey size, prey accessibility, and organoleptic preferences [45]. The results indicate that *V. zonata* larvae prefer *P. patulus* as the first food over *B. angularis*, switching on 11 dph to a larger prey (*M*. cf. *macrocopa*). The increase in mouth size is particularly accelerated during the first days after the mouth opening [72, 73] and delimits the dimensions of particle size that can be ingested. The range of accessible particle sizes increases as the larvae grow, gradually moving to larger and more energy-rich prey items [15, 45]. Alterations in the diet during the ontogenetic stage are associated with the prey size because the size of the preferred prey increases with predator size, following an allometric scaling theory [74]. The choice likely depends on factors such as prey biomass and energy provided [15, 45], as shown in the increasing consumption of probiotic-enhanced cladocerans when larvae reached a bigger size. During 11 dph, a change in preference for a larger zooplankton size took place, two factors might be influenced: (1) increasing mouth gape and (2) capturing effort and energy provided. Larvae tend to choose prey with a profitable relationship between energy gained and energy invested in capture [75, 76]. It was displayed during 11 dph when *P. patulus* and *M*. cf. *macrocopa* were suitable prey, but *M*. cf. *macrocopa* represents the best option in terms of this cost–benefit ratio.

Little information is available on feeding biology during the early stage of species from the genus *Vieja* and its rearing as a potential species produced for human consumption. During the 1990s, protein requirements, salinity tolerance, respiration rates, and stocking density were estimated for the closely related species *V. melanurus* (formerly *Cichlasoma synspila*; [77, 78, 79]). Olvera-Novoa et al. [79] studied the protein requirements in offspring of 45 dph (280 mg of weight) without information about the feeding of the larvae before the experiment. The protein requirement by *V. melanurus* is 40.81%. The lack of subsequent reports could represent a loss in interest in producing this species and is explained because, after 135 dph (4.5 months), *V. melanurus* juveniles reach 2.5 g, which represents more than double the time required by other economically important species, such as Nile tilapia (*O. niloticus* < 1.5 months; [80, 81]. Recent studies showed that commercial fish diets not only affected growth but also the larvae digestive tract morphology of *V. melanurus*, confirming that live food is a more suitable option [11]. Prey enhanced with probiotics gains a 12% increase in larval standard length and 13.5% in weight compared to the probiotic-free treatment. Larval body depth had a greater increase (16.2%) compared to the control treatment. It might be explained because of the morphological changes that occur during this transition stage since larvae have elongated bodies, while juveniles have deeper bodies [48]. The observed increase in growth and weight of *V. zonata* larvae fed with probiotic-enhanced prey is because probiotics act as growth promoters, inhibit the presence of pathogens, provide essential nutrients, help food processing and assimilation, increase tolerance to stress, stimulate the immune system, and even improve the quality of the environment [82, 83, 84]. In fish hatcheries, probiotics are traditionally supplied using live food as capsules, which ensures that probiotics are delivered directly to the digestive tract of the larvae. Using probiotics in the production of live food (bioenhance) confers: (1) an increase in prey numbers [28] and (2) carrying probiotics directly to larvae' digestive tract, conferring beneficial effects (aforementioned). Therefore, the 13.5% increase in the weight of *V. zonata* larvae in just 10 days (from day 5 to 15 dph) represents a fundamental reference in reducing production times, which would be reflected in more effective production with higher performance by reducing maintenance costs and production time. Although the current study relates to *V. zonata*, the results have practical applications for improving larval rearing in aquaculture. Probiotic-enhanced diets could optimize growth and survival, reducing production times and costs. By understanding the dietary shifts from endogenous to exogenous feeding, larviculture can better manage feeding strategies, ensuring larvae receive appropriate nutrition at critical stages.

More studies are needed on the early ontogenetic development of Middle American cichlids to better understand critical developmental stages and optimize rearing practices for enhanced survival and growth. Further research should explore the long-term impacts of early nutritional strategies, including different probiotic formulations, on the health and growth of larvae. Investigating the endocrine regulation of feeding and the ecological interactions of larvae can provide deeper insights into their feeding behaviors. Additionally, studies should assess the practical implications of probiotic use in commercial aquaculture, including the cost–benefit analysis of incorporating probiotics into live prey.

## 5. Conclusions

*V. zonata* larvae fed exogenously from day 5 dph when they still have a fraction of the vitellus. Larvae presented a mixed feeding type during the first days of post-hatching development, characterized by a period of endogenous feeding with the vitellus as the main source of food (0–5 dph), a mixed feeding period (5–7 dph), and an exogenous feeding period (starting from 7 dph). *V. zonata* has two critical feeding points during the first days after hatching: the first corresponds to the transition from endogenous to exogenous with a mixed period that ends with the exhausting of the vitellus; and the second is the shift from small prey (rotifers) to larger prey (cladocerans; 11 dph).

The preferred prey of larvae during the early days of development was *P. patulus* and later *M*. cf. *macrocopa*. The feeding regime of *V. zonata* larvae is presented. To achieve greater efficiency in the rearing of *V. zonata*, it is important to pay special attention to early feeding by offering rotifers and cladocerans starting from 5 dph. Probiotic-enhanced prey resulted in a 12% increase in SL, 13.5% in W compared to the control treatment (without probiotics), and a higher SGR. This work contributes to existing knowledge on the positive effects of probiotics by providing evidence on the probiotic properties by using them as live food bioenhancers. The study offers some important insights into the efficient use of probiotics, in addition to reducing the cost of their use by using live feed not only as probiotic carriers (bioencapsulation), improving the productivity and profitability of fish farming, therefore using probiotics would mean an investment and not an expense.

## Data Availability

The data that support the findings of this study are available from the corresponding author upon reasonable request.
